# Comparison of pre- and intraoperative findings in 35 cats and 60 dogs presenting with gastrointestinal signs

**DOI:** 10.3389/fvets.2025.1562792

**Published:** 2025-10-07

**Authors:** Sibylle M. Kneissl, Maria Laura Prüllage, Yasamin Vali, Jakub Vodnarek, Andrea Klang, Marlies Dolezal, Carina Strohmayer

**Affiliations:** ^1^Diagnostic Imaging, Clinical Centre for Small Animal Health and Research, Clinical Department for Small Animals and Horses, Vetmeduni, Vienna, Austria; ^2^Small Animal Surgery, Clinical Centre for Small Animal Health and Research, Clinical Department for Small Animals and Horses, Vetmeduni, Vienna, Austria; ^3^Centre of Pathobiology, Department of Biological Sciences and Pathobiology, Vetmeduni, Vienna, Austria; ^4^Platform for Bioinformatics and Biostatistics, Department of Biological Sciences and Pathobiology, Vetmeduni, Vienna, Austria

**Keywords:** dog, cat, diagnostic imaging, endoscopy, laparotomy, agreement, error, discrepancy

## Abstract

**Introduction:**

This retrospective study compared preoperative imaging and intraoperative findings in 35 cats and 60 dogs undergoing laparotomy.

**Objectives:**

The objective of this study was to evaluate the agreement between preoperative imaging and intraoperative findings in cats and dogs presenting with gastrointestinal signs.

**Methods:**

The medical archive of the teaching hospital was searched from 2021 to 2022 for dogs that presented with gastrointestinal signs. Only animals with preoperative imaging and laparotomy reports within 48 h were included and reviewed. The main imaging and surgical findings were extracted and classified as either ‘agreement’ or ‘no agreement’. Patients with incomplete or vague information were excluded. Additionally, the modality used for preoperative diagnosis (plain radiography, barium study, ultrasonography, computed tomography [CT], endoscopy) and the outcome (discharged, dead) were recorded. Agreement was assessed using Cohen’s kappa statistic. The sensitivity and pretest probabilities of preoperative imaging were calculated using the surgical report as the reference standard.

**Results:**

Agreement between the main imaging and surgical findings was achieved in 84 of 95 cases (88%). No agreement was noted in 11 patients (12%), of which 9 cases were false negative, and two cases were wrongly interpreted. Modalities used for preoperative imaging were ultrasonography (52%), plain radiography (42%), barium study (3%), CT (2%), and endoscopy (1%). Cohen’s kappa was 0 (*p* = not available) for sonography and 1 (*p* < 0.001) when using plain radiography. Sensitivity across all modalities, sonography, and plain radiography was 90.3, 81.6, and 100%, respectively, and corresponding pretest probabilities were 97.9, 100, and 95%. Eighty-two animals were discharged, and 13 patients either died or were euthanized.

**Conclusion:**

The clinical relevance of this work is providing evidence-based data on errors (no agreement) in preoperative imaging for patients with gastrointestinal disease. Radiography had significantly higher agreement with surgical findings compared to ultrasonography in dogs and cats presenting with gastrointestinal symptoms.

## 1 Introduction

Accurate preoperative diagnosis of gastrointestinal disorders in dogs and cats is essential for effective surgical decision-making. However, several studies have documented notable discrepancies between preoperative imaging and intraoperative findings in small animals with gastrointestinal disease. In a prospective study of 105 abdominal ultrasound examinations in dogs and cats, diagnostic errors were reported in 16.2% of cases (1). Retrospective analyses of patients with acute abdomen found agreement between ultrasonography and surgical findings in approximately 87%, with 13% of lesions missed, particularly gastrointestinal perforations or ruptured bile tract (2). A broader review of veterinary radiology suggests that, while only around 5% of findings are incorrect, there may be up to a 40% disagreement between antemortem and postmortem diagnoses. More recently, artificial intelligence has been proposed as a potential aid to reduce errors and improve diagnostic accuracy in veterinary imaging (3). Tracking diagnostic errors is important because it helps to identify the limitations of applied imaging methods and support evidence-based decision-making. By quantifying where and how errors occur - whether due to perception or cognition - clinicians can refine diagnostic protocols, reduce unnecessary surgeries and contribute to better outcomes for animals.

The objective of this study was to evaluate the agreement between preoperative imaging and intraoperative findings in dogs and cats presenting with gastrointestinal signs. The Kappa statistic, proposed by Cohen, was used to assess agreement. Additionally, to explore the accuracy of preoperative gastrointestinal diagnostic methods and surgical procedures, the sensitivity and pretest probabilities of imaging for major surgical findings were calculated.

## 2 Materials and methods

The electronic surgical logbook of the teaching hospital at Vetmeduni was searched for dogs and cats that had laparotomy performed from 2021 to 2022. Then medical records of patients presenting with gastrointestinal signs were reviewed in reverse chronological order to identify preoperative imaging reports (plain radiography, barium study, ultrasonography, computed tomography, endoscopy) performed within 48 h prior to laparotomy. Information filtered from the medical archive regarding these patients included signalment, imaging findings and diagnosis, and surgical findings. At Vetmeduni, radiology reports are generated in a clinical setting by either a senior not board-certified radiologist, a board-certified radiologist, or a supervised resident, mostly using a dual reading approach. The surgeon who performs the operation writes the surgical report. Imaging and surgical reports consisted of two fields, namely, findings and diagnosis, respectively, and observations and procedures performed. Surgical findings were classified as primary (main) or secondary based on the recorded observations as proposed (4, 5). The primary lesion was defined as the main surgical finding, while secondary lesions were categorized as any other related surgical findings. For instance, in an animal presenting with an intestinal mass and a foreign body, the intestinal mass would be considered the primary lesion, with the foreign body being categorized as a secondary finding. The main imaging and surgical findings were extracted from preoperative imaging and surgical reports (all fields) and classified for either “agreement” or “no agreement.” Additionally, the modality used for preoperative diagnosis and the outcome (discharged, dead) were recorded. This study included results from abdominal point-of-care ultrasound, but did not distinguish between point-of-care and traditional ultrasound findings because the latter was always performed before surgery. While some animals underwent more than one imaging procedure, the report that was decisive for laparotomy was the focus. Cases with incomplete or inconclusive surgery report were excluded. For example, a report that did not mention the site of a retrieved foreign body was considered incomplete. Similarly, a report of jejunal intussusception by the radiologist that was not observed during surgery was considered inconclusive, as the intestinal folding may have been influenced by general anesthesia. In cases where ‘no agreement’ was observed, two radiologists (C. S. and S. K.) and a pathologist (A. K.) came to a consensus. Findings described by the surgeon but not mentioned in the radiology report were classified as false negatives; findings not described by the surgeon and mentioned in the radiology report were classified as false positives. Imaging findings that were correctly described, but misinterpreted in the context of clinical reasoning, were classified as cognitive errors. Error classification was based solely on written reports and images were not re-evaluated. Statistical analyses were performed with R version 4.4.1. (R: A Language and Environment for Statistical Computing version 4.4.1, R Core Team [2024], https://www.R-project.org). Unweighted Cohen’s kappa was calculated with function kappa2 within the irr R package version 0.84.1 (6). Binary coded diagnostics from preoperative imaging and laparotomy was used to quantify pairwise agreement between imaging and surgery. A value of 1.0 indicates complete agreement and 0.0 indicates a level of agreement expected by chance alone (categories were: 0.0–0.2 poor agreement; 0.21–0.40 fair agreement; 0.41–0.60 moderate agreement; 0.61–0.80 good agreement; 0.81–1.0 excellent agreement). Sensitivity and pretest probability were calculated with custom R scripts. As other studies have not shown a relationship between body size of the animal and sensitivity to the main surgical lesions (4), we refrained from further statistical analysis of body size (i.e., differences between dogs and cats) in favor of sample size.

## 3 Results

Initially, 167 cases were retrieved. A total of 72 patients, including duplets and surgical reports with incomplete or vague information, were excluded. Thirty-five cats and 60 dogs were included in the study. Forty-four patients were females (27 spayed and 17 intact) and 51 were males (32 neutered and 19 intact). The mean body weight was 18.7 kg in dogs and 4.3 kg in cats. The mean age was 5.6 years (range 3.6 months to 15.5 years). The main breeds represented were European Shorthair (n = 20), Maine Coon (n = 6), Labrador (n = 5), Cocker Spaniel (n = 3), and French Bulldog (n = 3). Main surgical findings were classified as foreign body (n = 49), mass/nodule (n = 12), gastric dilatation-volvulus (*n* = 11), intussusception (*n* = 6), inflammation/necrosis (*n* = 5), ulcer (*n* = 3), herniation (*n* = 3), and small numbers of other pathologies (*n* = 4) such as gastric dilatation-volvulus/pyloric stenosis or postoperative complications (Table 1). Two patients had negative exploratory laparotomies. An agreement was reached between the preoperative and intraoperative findings in 84 out of 95 cases (88%). No agreement was noted in 11 patients (12%) (Table 2). Cohen’s kappa was 0 (*p* = not available) for sonography and 1 (*p* < 0.001) when using plain radiography. Sensitivity across all modalities, sonography, and plain radiography was 90.3, 81.6, and 100%, respectively, and corresponding pretest probabilities were 97.9, 100, and 95%. Modalities used for preoperative imaging were ultrasonography (52%), plain radiography (42%), barium study (3%), CT (2%) and endoscopy (1%). Eighty-two animals were discharged, and thirteen patients either died or were euthanized.

**Table 1:** Main surgical findings in dogs and cats with gastrointestinal disease, imaging modality used before surgery, agreement and type of error.

Pathological category	Cat	Dog	Modalities					Agreement		Type of error	
			BS	CT	EN	DR	US	Yes	No	FN	CE
Foreign body	20	29	2	1	1	22	23	47	2	2	0
Mass/nodule	5	7	0	0	0	0	12	9	3	3	0
Gastric dilatation-volvulus	0	11	0	0	0	11	0	11	0	0	0
Inflammation/necrosis	2	3	0	0	0	0	5	4	1	0	1
Intussusception	6	0	1	0	0	0	5	6	0	0	0
Ulcer	1	2	0	1	0	0	2	0	3	2	1
Herniation	0	3	0	0	0	3	0	3	0	0	0
Negative laparotomy	0	2	0	0	0	2	0	2	0	0	0
Others	1	3	0	0	0	2	2	2	2	2	0
Total	**35**	**60**	**3**	**2**	**1**	**40**	**49**	**84**	**11**	**9**	**2**

BS, barium study; CE, cognitive error; CT, computer tomography; DR, direct radiography; EN, endoscopy; FN, false negative; US, abdominal ultrasonography. The values that are highlighted in bold indicate the sum.

**Table 2:** Distribution of error according to preoperative imaging findings, intraoperative findings and postoperative histopathology.

ID	Error	Modality	Preoperative imaging findings (commented when CE)	Intraoperative findings	Species: Postoperative histopathology
23	CE	US	Presumed mechanical ileus (paralytic ileus caused by pancreatitis not considered)	Pancreatitis and paralytic ileus with gastric bleeding	Cat: Necrotizing pancreatitis and peritonitis
44	FN	US	Ascites, peritonitis, hepatobiliary disease, gastric dilatation, ileus, pancreataic edema, lymphadenomegaly	Suture dehiscence* following colectomy	Cat: Not performed
51	FN	US	Small bowel obstruction	Small bowel obstruction due to inflammation or neoplasia*	Dog: Intestinal adenocarcinoma
54	FN	US	Peritonitis, hepatitis, cholangitis, panreatic edema, lymphadenomegaly	Suture dehiscence* following colectomy	Dog: Not performed
68	FN	US	Enteritis	Foreign body*	Cat: Not performed
72	FN	US	Enteritis, presumed neoplastic small bowel infiltration, peritonitis	Perforated* neoplasia	Cat: Intestinal, transmural, large cell lymphoma, with resulting intestinal wall perforation
76	FN	US	Marked intestinal thickening suggesting either inflammation or neoplasia	Neoplasia and Briden ileus*	Cat: Intestinal adenocarcinoma with lymphogenic metastasis
80	FN	US	Enteritis	Foreign body*	Dog: Not performed
83	CE	CT	Presumed gastric ulcer (neither partial pyloric stenosis nor adhesions were considered)	Gastroduodenal adhesion and partial pyloric stenosis	Dog: Purulent gastritis with an eosinophilic component
84	FN	US	Pneumoperitoneum, enteritis, pancreatitis, peritonitis	Perforated ulcer*	Cat: Purulent, transmural enteritis/gastritis. The sample submitted was not sufficient to confirm the perforation.
97	FN	US	Pneumoperitoneum, peritonitis	Perforated ulcer*	Dog: Perforated ulcer

CE, cognitive error; CT, computer tomography; FN, false negative; US, abdominal sonography. Asterix (*) indicates an intraoperative finding that was missed during the preoperative imaging (false negative).

## 4 Discussion

The clinical relevance of this work is providing evidence-based data on errors in preoperative imaging for patients with gastrointestinal disease confirmed by laparotomy. An agreement between preoperative imaging and intraoperative findings was achieved in 84% of cases, with an overall error rate of 12%. This was predominantly due to false negatives (perceptive errors) and occasionally incorrect clinical reasoning (cognitive errors). These results are comparable to previous veterinary studies, which reported disagreement ranging from 13% in acute abdomen cases to 25% in routine ultrasonographic evaluations, most often small and difficult pathologies, involving gastrointestinal perforations, neoplasia, ulcer or suture dehiscence (2, 4). In contrast, radiography demonstrated excellent agreement in our cohort, confirming its value as a first-line modality in acute gastrointestinal presentations, particularly for foreign bodies and gastric dilatation-volvulus (7). Similar challenges have been documented in human radiology, where underreading, subsequent search miss, originally known as satisfaction of search (8), faulty reasoning, and location of the finding account for 42–9% of diagnostic disagreement (9). While perception errors, such as missed lesions, remain the most frequent in both veterinary and human practice, cognitive errors related to faulty reasoning or bias also contribute occasionally (10).

The performance of each imaging modality must be considered in light of its inherent limitations and potential sources of bias. Ultrasonography, although widely available and non-invasive, is highly operator-dependent and prone to perception errors, particularly when evaluating small or complex lesions obscured by intraluminal gas or adjacent structures, which may explain its relatively poor agreement in this study (Cohen’s kappa 0 and a sensitivity of 81.6%). Plain radiography, while demonstrating excellent agreement here (Cohen’s kappa = 1 and a sensitivity of 100%), may be biased by case selection, as it is most sensitive for radiopaque foreign bodies or gastric dilatation-volvulus, but far less reliable for subtle soft tissue lesions or mucosal disease (7). Barium studies, although performed infrequently in this study (3%), can improve delineation of gastrointestinal obstruction, yet their clinical utility is limited in emergencies due potential complications in patients with perforation (11). CT (2% of cases in our cohort) offers superior cross-sectional detail and higher sensitivity for certain lesions, such as ulcer, but availability, cost, and the need for general anesthesia may restrict its use in veterinary patients (12). Finally, endoscopy (1% of cases in our cohort) provides excellent visualization of mucosal pathology and is considered the gold standard for diseases such as inflammatory bowel disease (13), but requires general anesthesia and lacks the ability to assess extra-luminal or transmural processes. Taken together, these modality-specific factors introduce systematic biases into diagnostic accuracy, and clinicians must interpret preoperative imaging with careful consideration of both technical limitations and the clinical context.

This study reports very high pre-test probabilities for preoperative imaging. Understanding the interpretation of pretest probability is critical for interpreting the reported diagnostic performance. Pretest probability reflects the likelihood of disease before diagnostic testing. In the present cohort, values were exceptionally high for all imaging modalities (97.9% overall; 100% for ultrasonography; and 95% for plain radiography). This can be explained by the strict inclusion criteria, as only animals that underwent laparotomy within 48 h of imaging were evaluated. This created a population with a very high likelihood of needing surgery for gastrointestinal disease. This selection bias inevitably inflates predictive values, particularly the positive predictive value (PPV). While this reflects real-world conditions in referral hospitals, it does not translate to general practice populations, where disease prevalence is lower, and pretest probability is consequently reduced. Similar patterns are seen in human radiology: pretest probabilities are high in acute care imaging (e.g., suspected bowel obstruction), yielding strong PPVs. However, in screening contexts (e.g., mammography or CT lung screening), pretest probability is low, and PPVs may drop to 20–40% despite high test sensitivity (14–17). Therefore, the high pretest probabilities observed here reflect referral bias inherent to the study design and should be interpreted as context-specific rather than generalizable indicators of modality performance.

Elizabeth A. Krupinski has extensively studied how image quality affects a radiologist’s diagnostic accuracy (18, 19). Variations in image quality, such as low resolution, inadequate contrast, or suboptimal imaging protocols affecting spatial resolution, noise and contrast, are critical to reduce errors. For example, abdominal ultrasound is an unreliable indicator of the severity of pancreatitis (20), as demonstrated by the case of acute pancreatitis in this study that resulted in a cognitive error. CT scanning has a sensitivity of 67–93% for detecting gastrointestinal ulceration (11). In another case in this study, minimal wall changes in a patient with a history of perforated gastroduodenal ulceration led to a similar cognitive error. To enhance diagnostic reliability, the veterinary radiology community could focus on improving patient, human, and systemic factors in a comprehensive strategy.

In this study, the relatively low number of mistakes can be explained by a safe learning culture at Vetmeduni. We read images in a four-eyes-principle and many times dual reading (i.e., looking at images together). Two well established methods to foster clinical reasoning among clinicians are structured patient-orientated interdisciplinary rounds with persons participating having communication skill confidence (21) morbidity and mortality (M&M) rounds (22). M&M rounds are structured clinical meetings where the interdisciplinary veterinary team reviews cases with adverse outcomes, such as complications, unexpected deaths, or diagnostic/surgical errors. The purpose is not to assign blame, but to analyze errors, identify contributing factors, and extract lessons that can improve future practice. In fact, this study was presented at an M&M round and represents an attempt to switch from the lens of a radiologist to the lens of a small animal surgeon. Brookfield (23) considers reflection to be an event through four lenses – those of oneself, students (or patients), colleagues, and scientists. Switching the perspective is empowerment for both disciplines. It improves communication, problem solving, interdisciplinary communication, quality of care, and patient outcome. In this study, many cases had to be excluded because of incomplete or inconclusive surgery reports. Therefore, one action implemented in the corresponding M&M round was periodic content check of surgery reports (with a review focus on entity and location observed). Moreover, morning rounds include dedicated imaging training for staff focused on lesions, which are most frequently seen.

The excellent agreement between plain radiography and laparotomy results in this study can be partially explained by possible knowledge of the surgical report at the time of writing the report, as could have been the case after emergency and weekend services. The other limitation was the high pretest probability, suggesting a bias in patient sampling when selecting from the medical archive. Moreover, sampling and exclusion bias may compromise the validity of the research findings. Patients were not randomly selected but chosen in reverse order from three different retrieve electronic lists. Some patients had to be excluded due to incomplete or inconclusive surgical reports. Finally, specialist discrepancies in the medical archive complicated the retrospective analysis and resulted in failure to classify two patients. For example, in one case, the presumed diagnosis switched from gastrointestinal bleeding to hemorrhagic gastroenteritis without adding a rationale.

In conclusion, this study revealed excellent agreement between plain radiography and laparotomy results in dogs and cats with gastrointestinal disease, and poor agreement between sonography and laparotomy. This can be partially explained by a high proportion of relatively simple cases such as foreign bodies or gastric dilatation-volvulus when applying plain radiography. In agreement with other studies (1, 3, 4) small and difficult pathologies, such as ulceration or suture dehiscence, were missed most frequently using sonography. Thus, the error rates observed in this veterinary cohort not only reflect modality-specific limitations but also align with broader trends in radiology, emphasizing the need for structured error analysis, dual reading, and interdisciplinary review to enhance diagnostic accuracy and reduce adverse outcomes. To further evaluate the accuracy of preoperative imaging, prospective studies would be beneficial.

## Author’s note

Parts of this manuscript were presented at the ACVR Annual Scientific Meeting; OCT 30-NOV 2, 2024; Norfolk, VA, United States, and received the Diagnostic Imaging Award: Non-ACVR Resident.

## Data Availability

The raw data supporting the conclusions of this article will be made available by the authors, without undue reservation.
